# Comparative genomics reveals *Dehalogenimonas* genome dynamics and evolutionary trajectories toward organohalide respiration

**DOI:** 10.1128/aem.00579-26

**Published:** 2026-06-03

**Authors:** Yiru Cui, Xiuying Li, Xiaocui Li, Jingjing Wang, Huijuan Jin, Ke Shi, Jun Yan

**Affiliations:** 1 Key Laboratory of Pollution Ecology and Environmental Engineering, Institute of Applied Ecology, Chinese Academy of Sciences 74763 , Shenyang, Liaoning, China; 2 University of Chinese Academy of Sciences 74519, Beijing, China; 3 Provincial University Key Laboratory for Risk Prevention and Control of Emerging Contaminants, Nanjing Normal University 12534, Nanjing, Jiangsu, China; 4 School of Environment, Nanjing Normal University 12534, Nanjing, Jiangsu, China; Shanghai Jiao Tong University, Shanghai, China

**Keywords:** organohalide-respiring bacteria, *Dehalococcoidia*, reductive dehalogenase, genome evolution, *rdh *genomic island

## Abstract

*Dehalogenimonas* spp. specialize in organohalide respiration, a metabolism that features reductive dehalogenase (RDase) as the key catalyst, yet their evolutionary history and adaptation to halogenated substrates are largely unknown. Here, we surveyed the respiratory gene modules in *Dehalogenimonas* and constructed a pangenome for this genus. Comparative analyses revealed a genus-wide trend of genome streamlining, marked by contraction of gene families for general cellular functions, alongside preferential expansion of RDase-coding *rdhA* genes. Pairwise collinearity analysis showed poorly conserved gene order, indicating extensive genome rearrangements during the diversification of *Dehalogenimonas* lineages. Despite such genomic plasticity, all *Dehalogenimonas rdhA* genes assigned a definitive function are organized into operons positioned immediately adjacent to a two-component regulatory system, indicating that their transcription is tightly coupled to environmental signal sensing. We also identified an *ssrA*-associated genomic island carrying the signature *dcpA*, providing strong evidence for a role of mobile genetic elements in facilitating the spread of dihaloelimination potential across *Dehalogenimonas*. Selection pressure analyses further indicated that *dcpA* has been shaped by episodic positive selection at specific codon sites, indicative of adaptive fine-tuning, whereas purifying selection across many codon sites removes deleterious mutations and preserves core RDase catalytic function. Collectively, these streamlined, mosaic genomes provide an integrated evolutionary view of metabolic specialization in *Dehalogenimonas*.

**IMPORTANCE:**

Organohalide-respiring bacteria (OHRB) contribute to the cleanup of persistent halogenated contaminants in subsurface environments. Members affiliated with the genus *Dehalogenimonas* represent a distinct OHRB phylotype and have attracted growing attention for their ability to detoxify a broad range of organohalides, including the carcinogen vinyl chloride. Compared with other well-characterized OHRB, the genome architecture and evolutionary history of *Dehalogenimonas* spp. remain poorly resolved at the genus scale. Here, we address this knowledge gap by establishing the first comparative genomic framework for *Dehalogenimonas*. Our analyses reveal genome streamlining associated with specialization in organohalide respiration across the genus. We also identify conserved mechanisms that govern the acquisition and transcriptional control of *Dehalogenimonas* reductive dehalogenase genes. Together, this work sheds light on how *Dehalogenimonas* lineages evolve, acquire, and regulate essential respiratory components, providing a foundation to better understand their divergence and guide the deployment of these keystone OHRB in bioremediation practices.

## INTRODUCTION

Organohalide-respiring bacteria (OHRB) are taxonomically diverse, strict or facultative anaerobes adapted to using hazardous halogenated compounds as terminal electron acceptors (1). Central to this energy-conserving process, termed organohalide respiration, is reductive dehalogenase (RDase), a cobamide-dependent, iron-sulfur enzyme able to cleave carbon-halogen bonds and yield less toxic and/or more biodegradable products (2). OHRB play indispensable roles in natural attenuation and engineered bioremediation of anthropogenic organohalides, many of which are widespread subsurface contaminants (1). The genus *Dehalogenimonas*, within the class *Dehalococcoidia*, is a relatively recent addition to known organohalide-respiring phylotypes (3) and has since been frequently detected in contaminated ecosystems worldwide (4–7). Four *Dehalogenimonas* species, *D. lykanthroporepellens*, *D. alkenigignens*, *D. formicexedens*, and *D. etheniformans*, are formally described (3, 8–10), along with several unclassified *Dehalogenimonas* populations enriched from a variety of environmental habitats (11–13). While most *Dehalogenimonas* spp. grow exclusively via dihaloelimination (i.e., removing adjacent halogen substituents to form an alkene) of vicinally chlorinated C2–C3 alkanes, including 1,2-dichloroethane and 1,2-dichloropropane (1,2-DCP), some strains, such as *D. etheniformans* GP (10) and *Dehalogenimonas* sp. DCF (13), are also capable of respiring a chloroethene or chloroaromatic. The ability of *D. etheniformans* GP to metabolize the carcinogen vinyl chloride (VC) to ethene is of particular significance, as effective detoxification of VC under anoxic conditions was previously attributed solely to *Dehalococcoides mccartyi* (*Dhc*), another *Dehalococcoidia*-affiliated taxon.

Sequenced *Dehalogenimonas* genomes range from 1.7 to 2.1 Mbp in size, and each harbors 19–52 non-identical, putative RDase catalytic subunit-coding *rdhA* genes, constituting some of the largest *rdhA* repertoires reported for any known OHRB (10, 14–16). Despite such great dehalogenation potential, only three *rdhA* from *Dehalogenimonas* have been functionally characterized to date. Specifically, *dcpA* encodes the 1,2-DCP RDase DcpA, whereas the chloroethene RDase genes, *tdrA* and *cerA*, have been identified in *Dehalogenimonas* sp. WBC-2 (15) and *D. etheniformans* GP (10), respectively. The involvement of horizontal gene transfer in *Dehalogenimonas rdhA* acquisition is evidenced by the localization of *tdrA* within a discrete region that resembles the *Dhc* VC-dehalogenating genomic island (15). Regarded as the signature *rdhA* of *Dehalogenimonas*, *dcpA* was first identified in *D. lykanthroporepellens* BL-DC-9 and its widespread occurrence across *Dehalogenimonas* genomes is in line with the genus-wide dihaloelimination capacity (8, 9, 12). Notably, *dcpA* is also present in a few *Dhc* strains but has not been detected outside *Dehalococcoidia* (17). It remains unclear whether the distribution of *dcpA* reflects retention from an ancestral gene pool or recurrent intra-lineage gene exchange within a narrow subset of *Dehalococcoidia*. Beyond these *rdhA* features, several additional genomic characteristics have been reported for *Dehalogenimonas*. These include a very limited number of RDase membrane-anchoring *rdhB* genes (i.e., three to six genes per *Dehalogenimonas* genome) relative to their much larger *rdhA* repertoires (10, 14–16), and the presence of osmoprotectant biosynthesis genes involved in adaptation to fluctuating salinity (18, 19).

Prior studies have focused mainly on the phenotypic traits and dehalogenation capabilities of *Dehalogenimonas* spp., with limited knowledge of their genome architecture and evolutionary patterns. Here, we performed comparative genomic analysis to delineate the key respiratory gene modules and to resolve genome organization and conservation across the genus *Dehalogenimonas*. We revealed substantial reduction in *Dehalogenimonas* genome content and estimated lineage divergence times to place these traits in an evolutionary context. Detailed analyses further uncovered regulatory elements implicated in transcriptional control of *Dehalogenimonas rdhA* genes. We also identified mobile genetic elements that may facilitate *dcpA* acquisition and dissemination. Collectively, these results establish a genomic framework that links organohalide respiration-related genetic modules, regulatory potential, and evolutionary trajectories in *Dehalogenimonas*.

## RESULTS AND DISCUSSION

### Key metabolic and genetic features in *Dehalogenimonas*

**Fig 1:**
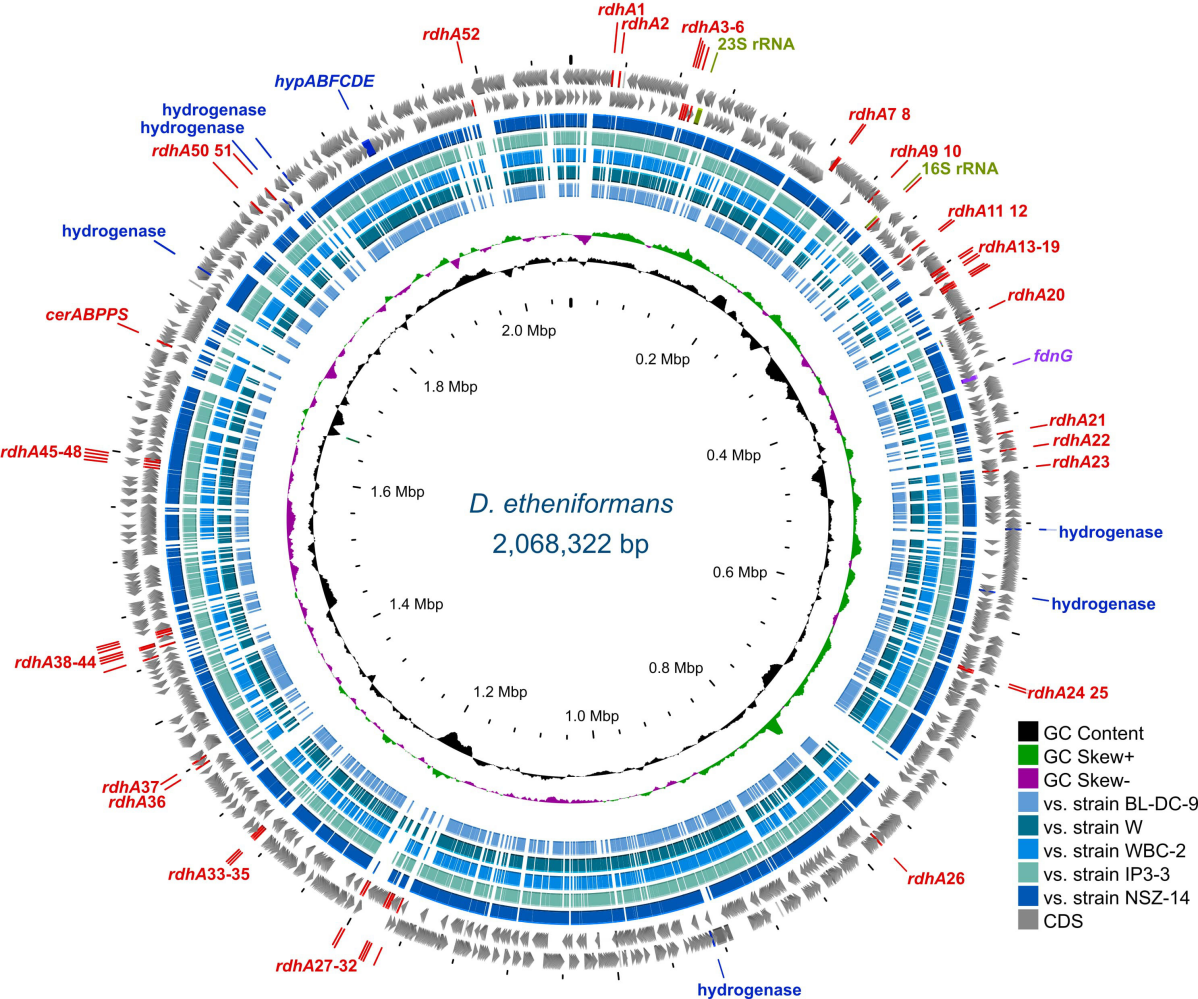
Circular map of the complete genome of *Dehalogenimonas etheniformans* GP. Rings from inside to the outside are as follows: ring 1, genomic coordinates; ring 2, GC content; ring 3, GC skew; rings 4–8, BLASTN hits (with an *E*-value cutoff of 10^−4^) against the genomes of *D. lykanthroporepellens* BL-DC-9 (blue-gray), “*Candidatus Dehalogenimonas loeffleri*” W (blue-green), *Dehalogenimonas* sp. WBC-2 (light blue), *D. alkenigignens* IP3-3 (scaffold) (light green), and *D. formicexedens* NSZ-14 (dark blue); rings 9 and 10, (gray) coding regions on the strain GP genome. The 52 predicted *rdhA* homologs, including *cerA*, are highlighted in red on the corresponding strand and numbered 1–52 according to their clockwise positions from the origin of replication.

A total of nine *Dehalogenimonas* genomes were retrieved from the NCBI RefSeq database for comparative analyses, and their basic statistics were summarized in Table 1. Figure 1 presents a circular BLAST-based comparison of representative *Dehalogenimonas* genomes, illustrating genus-level genome organization and conserved gene orders. This visualization reveals that *Dehalogenimonas rdhA* genes are dispersed across multiple loci rather than being confined to specific genomic regions. Such a pattern contrasts with that observed in *Dhc* genomes, in which most *rdhA* genes are located within high-plasticity regions flanking the origin of replication (20, 21). These genomes lack alternative terminal electron-accepting pathways (e.g., nitrate or sulfate reduction), as well as genes involved in some common bacterial functions, including peptidoglycan synthesis and environmental stress adaptation (e.g., tolerance to acidic or alkaline conditions). Nitrogenase genes (*nif*) are rarely detected in *Dehalogenimonas*, with strain WBC-2 being the only genome found to carry a partial *nif* gene set, including *nifH* (locus # DGWBC_0610), *nifD* (DGWBC_0607), and *nifK* (DGWBC_0606). Nevertheless, the absence of essential FeMo-cofactor biosynthesis genes *nifB* and *nifV* suggests that nitrogen fixation is unlikely to be functional in strain WBC-2. By comparison, *Dhc* strain 195 possesses a complete *nif* operon (locus # DET1151-1158) together with a distally located *nifV* (DET1614) (22), and has been experimentally shown to reduce dinitrogen to ammonia (23); however, studies to date suggest that this capability is limited to certain Cornell-clade *Dhc* strains (24, 25). Together, these observations indicate that nitrogen fixation is an uncommon trait in *Dehalococcoidia* and may represent an ancestral feature of *Dhc* retained through the evolutionary transition to an organohalide-respiring lifestyle. Cobamides, the vitamin B_12_-family coenzymes, are essential for RDase catalysis (26), yet the analyzed *Dehalogenimonas* genomes, except for strain NSZ-14 and strain W, lack most of the genes required for the biosynthesis of a precorrin (i.e., cobamide precursor). Instead, these genomes consistently encode functions for cobamide salvaging and downstream processing, including the *btuFCD* transport system and cobamide remodeling/assembly modules (e.g., *cobA*, *cbiZ*, *cbiB*, *cobD,* and *cobTSCU*) (27). Most of these genes are clustered within a 10- to 12-gene locus, as observed for HX448_06240-06295 in strain GP and Dform_00895-00904 in strain NSZ-14. The precorrin biosynthesis *hem* and *cbi* genes present in strain NSZ-14 and strain W are located outside this locus. Despite analysis with multiple bioinformatic tools, we found no convincing evidence for horizontal acquisition of these genes, suggesting that they were more likely maintained through vertical inheritance. Nevertheless, no *Dehalogenimonas* strain has yet been experimentally demonstrated to be capable of synthesizing cobamides *de novo*. In summary, these features indicate that *Dehalogenimonas* spp. are generally corrinoid auxotrophs and likely rely on exogenous cobamide supply to sustain dehalogenation activity. The conserved genomic arrangement may reflect the evolutionary retention of cobamide salvaging genes as an ancient adaptive strategy.

**TABLE 1:** Summary of basic features of the *Dehalogenimonas* genomes analyzed in this study

	GP	NSZ-14	BL-DC-9^*a*^	IP3-3*^b^*	BRE15M^*b*^	4OHTPN	W	WBC-2^*a*^	THU2*^b^*
Size (Mbp)	2.07	2.09	1.69	1.85	1.65	1.75	1.77	1.73	1.84
CDSs	2,037	2,115	1,659	1,891	1,698	1,772	1,770	1,721	1,874
G+C (mol%)	51.9	54.0	55.0	55.9	56.3	55.9	52.6	49.2	55.1
rRNAs	3	3	3	3	3	3	3	3	3
tRNAs	47	48	47	47	48	47	47	48	48

^*a*^ RefSeq records of strain BL-DC-9 and strain WBC-2 have been suppressed by NCBI.

^*b*^ The genomes of strain IP3-3, strain BRE15M, and strain THU2 are not closed and comprise 2, 18, and 35 contigs, respectively.

**Fig 2:**
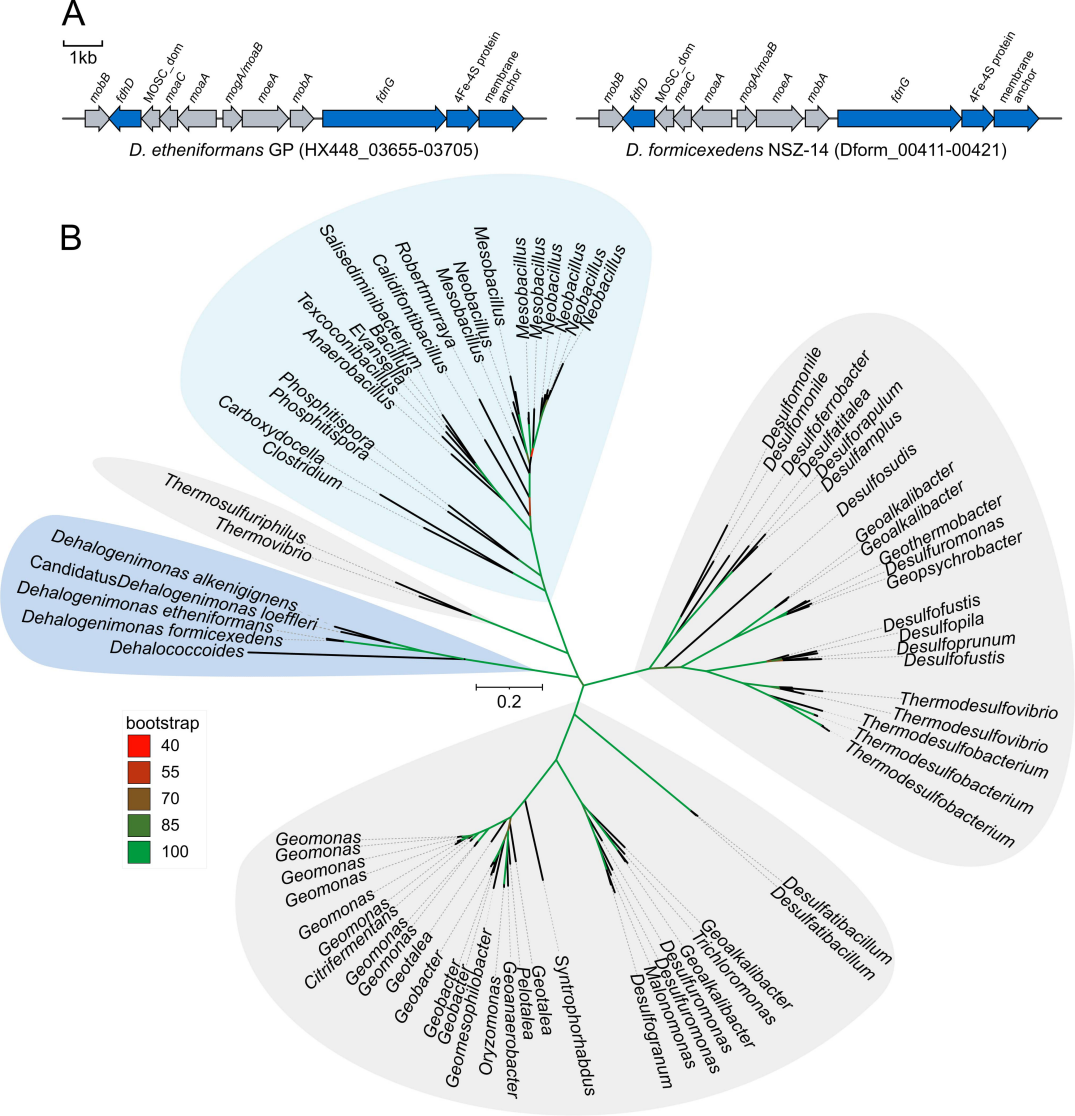
Gene organization of the *fdn* cluster in *Dehalogenimonas* and phylogenetic tree of FdnG proteins. (**A**) Illustration of the *fdn* gene cluster in *D. etheniformans* GP and *D. formicexedens* NSZ-14. Blue: formate dehydrogenase genes; gray: *bis*-MGD biosynthesis genes. *mobB*, MGD biosynthesis accessory protein B gene; MOSC_dom, MOSC domain-containing molybdenum cofactor sulfurase-like protein CDS; *moaC*, cyclic pyranopterin monophosphate synthase gene; *moaA*, GTP 3',8-cyclase gene;* mogA*/*moaB*, molybdopterin-adenylate intermediate synthesis gene; *moeA*, molybdopterin molybdotransferase gene;* mobA*, *bis*-MGD biosynthesis gene; *fdhD*, formate dehydrogenase accessory sulfurtransferase gene. (**B**) Maximum likelihood phylogenetic tree of 100 FdnG homologs from the phyla *Chloroflexota*, *Thermodesulfobacteriota*, and *Bacillota*. The tree was constructed using the *D. etheniformans* GP FdnG (WP_102331337) and its top BLAST hits from NCBI RefSeq Selected Proteins database, including an FdnG-like protein from *Dhc* strain 195 that lacks formate-oxidizing activity but shares the highest sequence identity to *Dehalogenimonas* FdnG. Branch colors represent the ultrafast bootstrap support values, shown as percentages based on 1,000 resamplings and mapped according to the adjacent gradient scale. Dark blue shaded, *Chloroflexota*; gray shaded, *Thermodesulfobacteriota*; and light blue shaded, *Bacillota*. Genus names are labeled along the tree, with overlapping identical labels omitted for clarity.

All *Dehalogenimonas* genomes encode three types of hydrogenases in common (Table S1), a periplasmic Hup-type hydrogen uptake hydrogenase, a cytoplasmic Vhu-type hydrogenase, and a coenzyme F_420_-reducing hydrogenase homolog. As resolved in strain BRE15M, the Hup-type hydrogenase assembles with an RDase, the associated RDase anchor protein, and a formate dehydrogenase-N (FDH-N) complex to form the central part of the multi-enzyme respiratory chain (28). The Hup-type hydrogenase is the only periplasmic hydrogenase in *Dehalogenimonas* and likely acts as the primary electron-entry module of the respiratory chain with H_2_ as the electron donor. First structurally characterized in *Escherichia coli*, FDH-N is a membrane-bound, molybdenum-dependent system that catalyzes formate oxidation to CO_2_ (29). This enzyme complex is encoded by the *fdnGHI* operon, where *fdn* denotes FDH-N; *fdnG*, *fdnH*, and *fdnI* specify the catalytic *α* subunit (i.e., FdnG), the [4Fe-4S]-containing electron-transfer *β* subunit, and the *b*-type cytochrome *γ* subunit involved in electron transfer and membrane anchoring, respectively (30). Across all analyzed *Dehalogenimonas* genomes, the *fdn* operon is conserved and retains the complete set of FDH-N genes in an organization similar to that of *E. coli* K-12. The catalysis of formate oxidation by FdnG requires both an active-site selenocysteine residue and a molybdenum cofactor (Moco). Consistent with this enzymatic constraint, *Dehalogenimonas* FdnG proteins contain a selenocysteine residue, and all analyzed genomes encode the *selCDAB* operon required for selenocysteine biosynthesis and incorporation (e.g., HX448_01345-01360 in strain GP; Table S2) (9). Genes required for biosynthesis of the Moco, *bis*-molybdopterin guanine dinucleotide (*bis*-MGD), including the *bis*-MGD precursor synthesis genes *moaA* (i.e., GTP 3′,8-cyclase gene) and *moaC* (i.e., cyclic pyranopterin monophosphate synthase gene), and *moeA* (i.e., molybdopterin molybdotransferase gene), are also commonly found in *Dehalogenimonas* genomes but absent from sequenced *Dhc* genomes. For strain GP and strain NSZ-14, the Moco biosynthesis genes co-localize with the *fdnGHI* operon and the *bis*-MGD sulfuration and insertion gene *fdhD*, together forming a compact 11-gene cluster (HX448_03655-03705 and Dform_00411-00421, respectively) (Fig. 2A). In other *Dehalogenimonas* genomes, the Moco biosynthesis genes are located distantly from the *fdnGHI* operon. Notably, in strain IP3-3, strain 4OHTPN, and strain W, a Moco riboswitch was identified directly upstream of *moaA* and *moaC* (locus # DEALK_13370-13380, ABV300_05450-05455, and V8247_08550-08555, respectively), which encode enzymes that initiate Moco production by converting 5′-GTP to cyclic pyranopterin monophosphate (31). Such a genomic arrangement likely allows the biosynthesis pathway to be regulated in response to intracellular molybdenum levels, avoiding unnecessary energy costs when Moco pools are sufficient.

Prior biochemical efforts suggest that *Dehalogenimonas* FdnG functions analogously to *Dhc* OmeA (32), a respiratory component proposed to mediate electron transfer from Hup-mediated H_2_ oxidation to RDase (28). These genetic and biochemical lines of evidence support a bifunctional role for *Dehalogenimonas* FDH-N, which can act downstream of Hup to relay H_2_-derived electrons to RDase, or serve as an electron-entry module when formate is available. It is worth noting that the FdnG proteins of *Dehalogenimonas* share only ~50% sequence identity with their 100 closest BLASTP homologs in the NCBI RefSeq Selected Proteins database, all of which are encoded by members of the phyla *Thermodesulfobacteriota* and *Bacillota*, and only 36.4–41.9% sequence identity with *E. coli* K-12 FdnG. *Dehalogenimonas* FdnG proteins form a distinct, well-supported clade as displayed in the maximum-likelihood phylogeny (Fig. 2B). Their pronounced divergence from canonical FdnG proteins suggests lineage-specific functional adaptation and may explain the proposed dual electron-transfer role of FDH-N in *Dehalogenimonas*.

### *Dehalogenimonas* pangenome

Due to a contamination warning related to sequence quality, the *D. lykanthroporepellens* BL-DC-9 genome was excluded from pangenome construction. OrthoMCL-based clustering was performed using protein-coding sequences (CDSs) from the remaining eight *Dehalogenimonas* genomes. A total of 13,248 homologs from the 14,733 analyzed CDSs were classified into 2,340 homolog families. The numbers of analyzed CDSs, genes in homologs, genes in singletons, and homolog families (i.e., clusters) for each genome are summarized in Table S3. The core gene set comprises 7,810 CDSs grouped into 969 protein families, representing 53% of the CDSs conserved among these *Dehalogenimonas* genomes (Fig. S1 and Data Set S1). The number of homologous genes shared among genome pairs ranges from 1,822 to 1,950 (Fig. S2), with the largest shared gene set (1,950 orthologs) observed between strain GP and strain NSZ-14. The most conserved gene families across analyzed *Dehalogenimonas* genomes are *rdhA* and genes involved in some basic cellular functions. Processes encoded by these genes include carbon fixation (e.g., WP_102330414), amino acid synthesis (e.g., WP_226846917), iron-sulfur cluster assembly (e.g., WP_102331813), ion transmembrane transport (e.g., WP_102331205), metallopeptidase activity (e.g., WP_102330885), coenzyme A biosynthesis (e.g., WP_102330118), and tRNA modification (e.g., WP_102330385). A total of 128 clusters consisting of 258 inferred proteins are shared exclusively by strain GP and strain NSZ-14 (Fig. S1, row 2), which have the largest genomes (i.e., ~2.1 Mbp) among sequenced *Dehalogenimonas*. These enzymes are inferred to participate in core cellular processes, including phospholipid biosynthesis (e.g., WP_102331436 and WP_076003479), NAD(*P*)H-dependent FMN reduction (e.g., WP_102331089 and WP_076004809), formate/nitrite transport (e.g., WP_264294057 and WP_076004073), and response to oxidative stress (e.g., WP_102331171 and WP_076003269). The 62 unique clusters shared among strain IP3-3, strain BRE15M, and strain 4OHTPN include enzymes involved in transcriptional regulation (e.g., WP_058439729), FtsZ-dependent cytokinesis (e.g., WP_133240184), tRNA modification (e.g., WP_065128750), phosphorelay signal transduction (e.g., WP_058438909), and pyridine nucleotide biosynthesis (e.g., WP_058439278), likely reflecting species-specific variations.

Strain GP and strain WBC-2 possess the highest numbers of singleton genes (307 and 295, respectively). An archaease (WP_102330077) was encoded solely in strain GP genome, which is predicted to be a chaperone involved in DNA or RNA metabolism by protecting enzymes against aggregation. Strain GP also harbors genes for Mrr (methyl-directed restriction) enzymes (WP_102331789 and WP_102330862), which are Type IV restriction endonucleases that recognize and cleave methylated foreign DNA, suggesting an enhanced defense against invasive genetic elements such as phages and plasmids. Since strain GP was isolated from grape pomace, a microbially complex environment, the presence of Mrr system genes may reflect an evolutionary response to phage predation pressure. This observation also aligns with the growing recognition that restriction systems function as critical barriers to phage infection and horizontal gene transfer in densely populated microbial ecosystems (33). The genome of strain WBC-2 contains numerous insertion sequence elements among its singleton genes, including two IS21 family transposase genes (DGWBC_0418 and DGWBC_0422) located near *tdrA*. This transposon load may facilitate genome rearrangement and *rdhA* recombination, potentially contributing to the diversification of *rdhA* genes in strain WBC-2. Nevertheless, most singleton genes in the *Dehalogenimonas* pangenome remain functionally uncharacterized, consisting mainly of hypothetical proteins and CDSs encoding domains with no assigned function.

### Lineage divergence and genome streamlining in *Dehalogenimonas*

**Fig 3:**
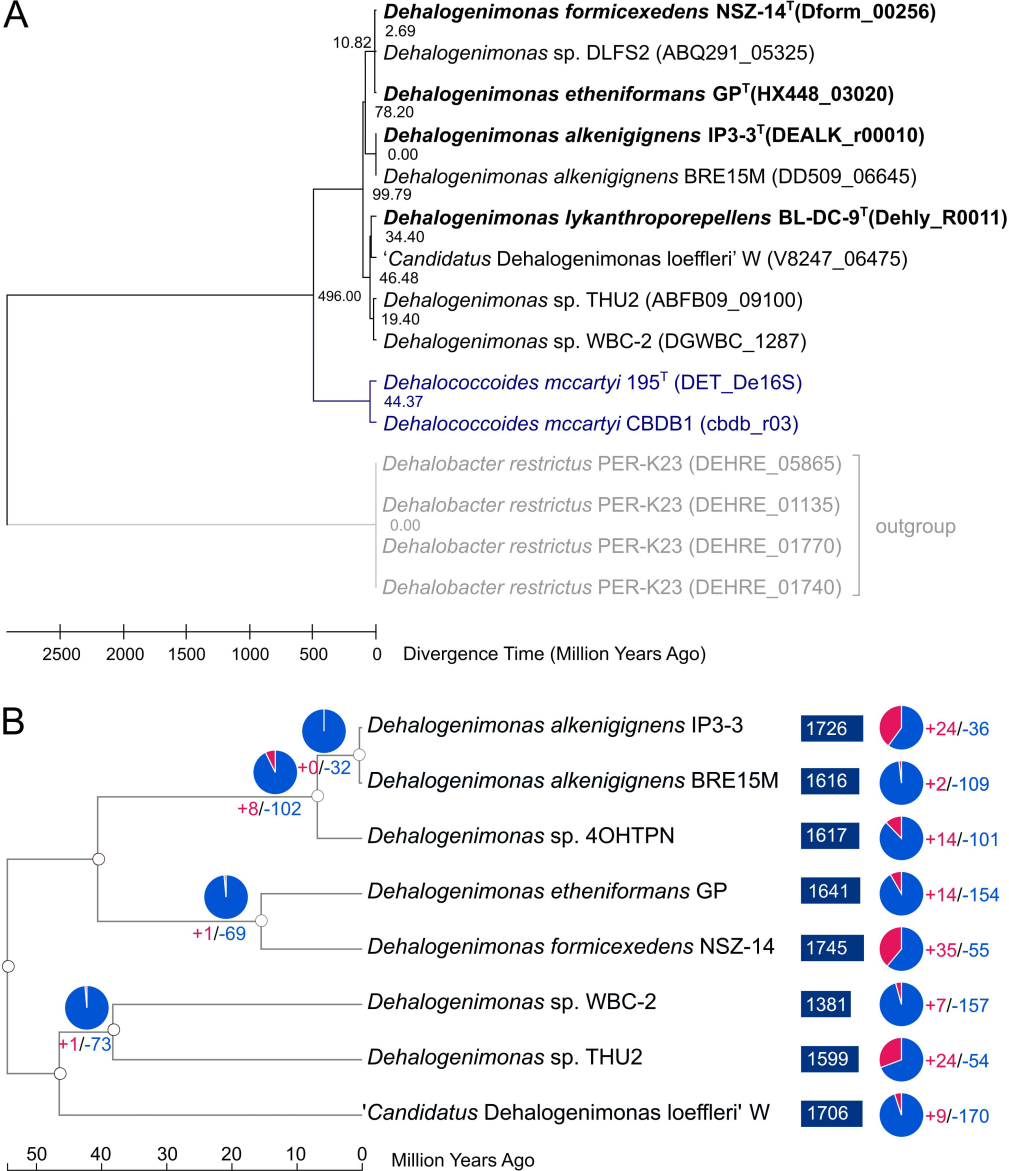
Divergence time estimation and gene family contraction and expansion in *Dehalogenimonas.* (**A**) Phylogeny and molecular clock analysis based on selected 16S rRNA gene sequences from *Dehalogenimonas* spp. and *Dhc*. *Dehalobacter restrictus* PER-K23 was used as the outgroup. Branch lengths represent the divergence times (mya) estimated using the RelTime method in Mega X. The reported divergence time of 496 mya between *D. lykanthroporepellens* and *Dhc* was used as the calibration point. (**B**) Gene family contraction and expansion in *Dehalogenimonas.* The number of orthologous gene families in each genome is shown in a horizontal bar graph, and the numbers of contracted (blue) and expanded (red) gene families are shown in the pie chart.

16S rRNA gene sequences were used for TimeTree analysis to evaluate the evolutionary time frame of *Dehalogenimonas* lineage divergence (Fig. 3A). While most *Dehalogenimonas* lineages diverged between 99.8 and 34.4 million years ago (mya), the speciation event separating *D. etheniformans* and *D. formicexedens* occurred much more recently, at approximately 10.8 mya. The TimeTree analysis also suggested that the common ancestor of *Dehalogenimonas* and *Dhc* diverged from other OHRB phylotypes during the Archean eon (4,000–2,500 mya) (Fig. S3). We then used the estimated divergence times as temporal scale references to examine gene family contractions and expansions. The analyzed genomes were shown to contain more contracted than expanded gene families (Fig. 3B), indicating that genome streamlining is a prevailing evolutionary pattern in *Dehalogenimonas*. In the case of strain GP, the contracted gene families are associated with transcriptional regulation, protein translocation, lipid-modifying glycosylation, cobamide biosynthesis, and cobalt ion transport, whereas the expanded ones include *rdhA* and those coding for iron-sulfur cluster assembly, cobamide precursor activation, and polysaccharide biosynthesis. The expansion of *rdhA* was also observed in strain NSZ-14, along with genes coding for cobamide salvaging/remodeling and ribonucleoside-diphosphate reductase (RNR). Strain W appears to have undergone the most extensive reductive evolution, as reflected by contraction in gene families responsible for general cellular functions, including DNA replication (i.e., RNR), phosphorelay signal transduction (i.e., phosphohydrolase), and carbohydrate metabolism (i.e., phosphoglucosamine mutase). The selective expansions of *rdhA* and iron-sulfur cluster assembly genes underscore the central role of organohalide respiration in driving *Dehalogenimonas* evolution. A detailed list of contracted and expanded gene families across the analyzed *Dehalogenimonas* genomes is provided in Data Set S2. Substantial reduction of *Dehalogenimonas* genomes reflects an evolutionary strategy in which nonessential functions are preferentially discarded to preserve cellular resources and reinforce metabolic specialization (i.e., organohalide respiration). The selective pressures driving such large-scale genome streamlining likely stem from the ecological niche occupied by *Dehalogenimonas*. These bacteria inhabit strictly anaerobic, resource-limited subsurface environments, where selection may favor the reduction of metabolic modules when essential resources, such as cobamides, are available through synergistic interactions rather than *de novo* biosynthesis.

### Low syntenic conservation across *Dehalogenimonas* genomes

We next performed collinearity analyses at the whole-genome scale to assess the extent of chromosomal rearrangements and genome plasticity within *Dehalogenimonas*. CDSs were compared across all pairwise genome combinations to identify collinear genes, and the contiguous regions formed by these collinear genes were defined as locally collinear blocks (LCBs). We anchored the analysis on the reference-quality genomes of the two type strains, *D. etheniformans* GP and *D. formicexedens* NSZ-14, and then projected LCB boundaries onto the remaining assemblies. Pairwise comparisons (Fig. S4) revealed limited synteny across *Dehalogenimonas* genomes. The highest degree of genome synteny was observed between strain GP and strain NSZ-14, as reflected by 2,880 of 4,083 genes (70.54%) mapping collinearly across only 24 LCBs and no evidence of large-scale insertions, deletions, or inversions. GP and WBC-2, the two *Dehalogenimonas* strains able to utilize a chlorinated ethene, were shown to share only 44.45% (1,661/3,737) genes in collinear arrangement across 70 LCBs. Limited synteny was also observed between strain GP and strain W (42.63%, 1,611/3,779 genes; 71 LCBs), whereas a higher proportion of collinear genes was detected between strain GP and strain IP3-3 (58.40%, 2,273/3,892 genes; 71 LCBs). The use of strain NSZ-14 genome as the reference yielded comparable results, identifying 70 LCBs with strain WBC-2 (44.46%, 1,684/3,788 genes), 76 LCBs with strain IP3-3 (64.42%, 2,540/3,943 genes), and 80 LCBs with strain W (47.49%, 1,819/3,830 genes). Pairwise genome comparisons among strain IP3-3, strain W, and strain WBC-2 recovered only 46.84–55.02% collinear genes (Data Set S1). Synteny between a *Dehalogenimonas* genome and a *Dhc* genome (represented by strain 195) was substantially lower, with the highest proportion of collinear genes reaching only 35.34%. Collectively, these results indicate pervasive genome rearrangements across the genus *Dehalogenimonas*. Notably, the high genome collinearity between strain GP and strain NSZ-14, together with their 99.5% 16S rRNA gene sequence similarity, supports the placement of this pair as the most closely related *Dehalogenimonas* lineages.

### Transcriptional regulation of *Dehalogenimonas rdhA*

**Fig 4:**
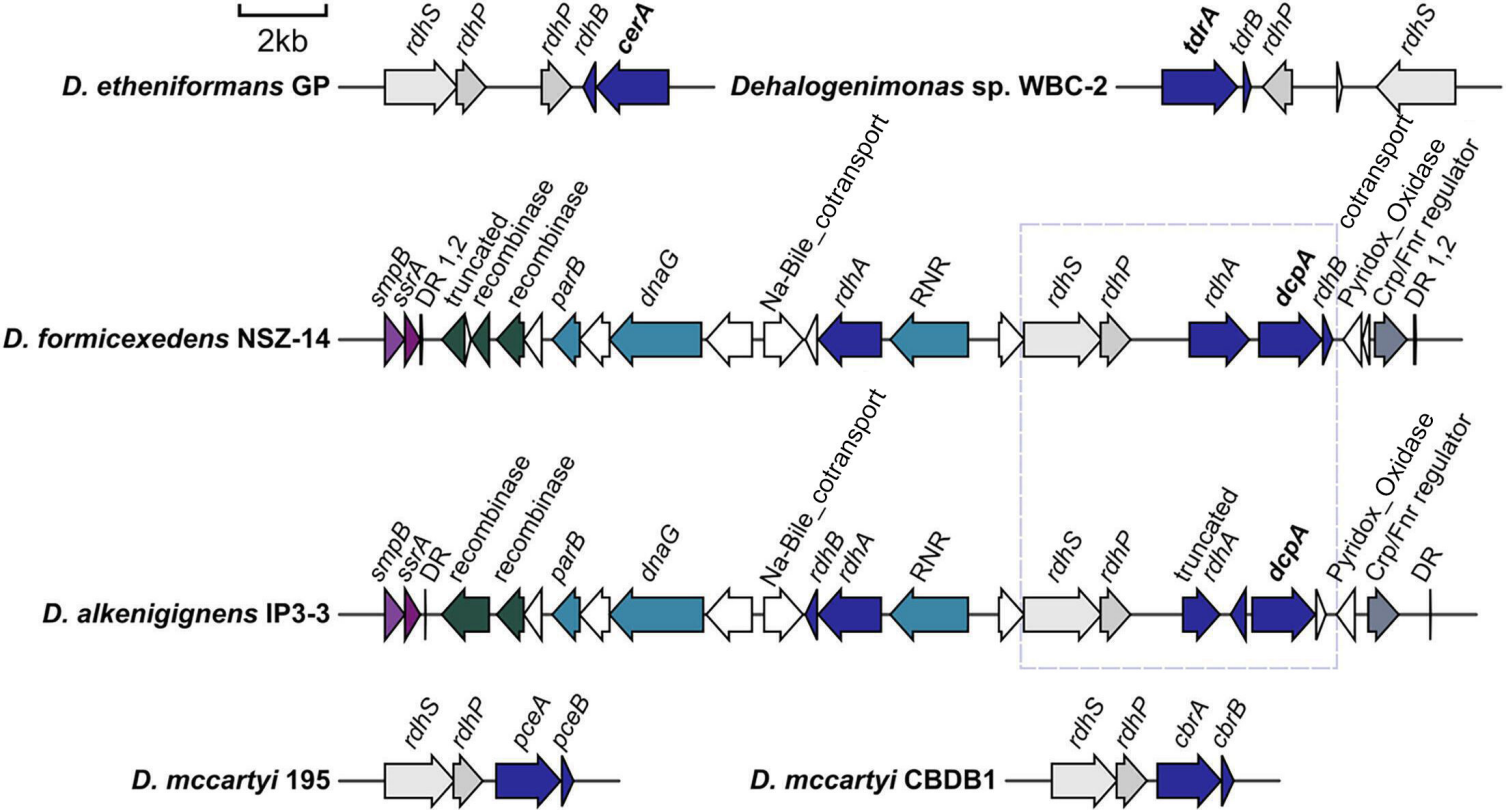
Comparison of *rdh* gene clusters in *Dehalogenimonas* and *Dhc* strain 195 (22) and *Dhc* strain CBDB1 (36). Corresponding locus tags are HX448_10000-10020 (GP), DGWBC_0411-0415 (WBC-2), Dform_01443-01467 (NSZ-14), DEALK_17010-17250 (IP3-3), DET0315-0319 (195), and cbdbA82-A85 (CBDB1). The *dcpA*-containing genomic islands in *D. formicexedens* NSZ-14 (1,384,297–1,407,259 bp) and *D. alkenigignens* IP3-3 (1,612,218–1,635,409 bp) are delineated by direct repeats (DRs) downstream of *ssrA*. The *rdhAB* genes are shown in dark blue, regulatory genes in gray, recombinase genes in dark green, DNA replication-associated genes in teal, and other genes in white. *smpB*, SsrA-binding protein SmpB gene; *parB*, chromosome-partitioning protein ParB N-terminal domain-containing protein CDS; *dnaG*, gene encoding a putative DnaG-type primase catalytic core-containing protein; Na-Bile_cotransport, sodium/bile acid cotransporter family protein CDS; RNR, ribonucleoside-diphosphate reductase; Pyridox_Oxase, pyridoxamine 5′-phosphate oxidase family protein; Crp/Fnr regulator, Crp/Fnr family transcriptional regulator gene. Unlabeled arrows indicate hypothetical proteins of unknown function. Note, the locus tag for *dcpB* in strain IP3-3 was removed in the latest genome annotation update; however, manual inspection using the RAST server identified a 228 bp *dcpB* immediately downstream of *dcpA*.

All functionally characterized *rdhA* are positioned immediately upstream of a ~160–200 bp *rdhB* (i.e., the *rdhAB* locus) (10, 15, 17). As predicted by InterPro, the anchor protein is highly hydrophobic, with two to three transmembrane helices and a positively charged cytosolic N-terminus. Except for the *dcpAB* (Dehly_1524-1525) in strain BL-DC-9, whose genome was flagged for contamination, *Dehalogenimonas rdhAB* loci with validated function (i.e., *dcpAB*, *tdrAB*, and *cerAB*) share an identical operon context. As shown in Fig. 4, each of these loci is located in close proximity to genes encoding a two-component regulatory system (TCS). The TCS module, consisting of a sensor histidine kinase (HK) and a response regulator (RR), represents a canonical prokaryotic signaling mechanism for transcriptional regulation (34). The HK senses various environmental stimuli, autophosphorylates, and transfers the phosphate group to its cognate RR, which typically possesses DNA-binding activity and can modulate gene expression (34). TCS-regulated *rdhA* transcription has been proposed in *Dhc* and *Sulfurospirillum multivorans* (22, 35–37), where the corresponding genes are designated *rdhP* (RR-encoding) and *rdhS* (HK-encoding) (38). The inferred *Dehalogenimonas* HKs and RRs share 27.4–36.2% amino acid identities with their homologs from *Dhc* and *Sulfurospirillum multivorans* (Tables S4 and S5). In each *Dehalogenimonas* genome, between 1 and 13 *rdhA* genes, in addition to *dcpA*, *cerA*, and *tdrA*, are consistently found in close proximity to an *rdhPS* cluster. Most reported HKs function as membrane-associated receptors with transmembrane segments and diverse input domains (39). In contrast, *Dehalogenimonas* HKs lack predicted transmembrane helices, a feature similar to those predicted in *Dhc* (35). This pattern suggests that signal perception primarily occurs in the cytoplasm rather than at the membrane interface, potentially through direct sensing of intracellular organohalide molecules or indirect interactions with redox-sensitive cellular components; however, this hypothesis remains to be experimentally validated.

Genome survey further suggests that *Dehalogenimonas rdhA* transcription is also under negative control by MarR-family regulators. MarR proteins typically repress basal transcription by binding target promoter regions and are released from DNA upon binding specific ligands or changes in redox-active metabolites (40). These regulators are characterized by a winged helix-turn-helix (HTH) motif that enables the dimer to recognize short palindromic sites in the target sequence (40). A *marR* gene, which encodes one of the MarR-family regulators, has been experimentally shown in *Dhc* strain CBDB1 to repress downstream *rdhAB* expression via direct binding of MarR to the *rdhA* promoter (41). In strain GP, five *rdhA* genes (locus # HX448_00330, HX448_03315, HX448_03490, HX448_07520, and HX448_09535) are each located downstream of and are divergently oriented to a *marR* homolog. One of these *rdhA* (HX448_03490) is uniquely distinguished by a short, 6-bp palindromic motif positioned ~20 bp upstream within its promoter region and by its co-localization with a *rdhB*. One or two *rdhA* genes positioned downstream of *marR* genes were identified in the genomes of other *Dehalogenimonas* strains, including NSZ-14 (locus # Dform_01599), IP3-3 (locus # DEALK_06310 and DEALK_07360), BRE15M (locus # DD509_04360 and DD509_04575), and W (locus # V8247_05215). In addition to TCS and MarR-family regulators, a CRP/FNR transcription factor (locus # Dform_02002 and DEALK_00320) was identified adjacent to an *rdhA* gene in strain NSZ-14 and strain IP3-3. It has been reported that the CRP/FNR superfamily regulators respond to a variety of effector molecules, including organohalides, by binding them via an N-terminal sensory domain and transmitting the signal to a C-terminal DNA-binding domain to activate transcription (42, 43). Prior studies have demonstrated that a CRP/FNR-family regulator can activate *Desulfitobacterium rdhA* transcription in the presence of 3-chloro-4-hydroxyphenylacetic acid (44, 45).

Among these systems, the TCS is expected to play a major role in transcriptional control since it is the only regulatory element consistently located in the immediate vicinity of *dcpA*, *tdrA*, and *cerA*. In a previous study on strain BL-DC-9, transcripts of several RR genes were detected together with those of their cognate *rdhA* (46). Future studies should verify the regulatory role of the TCS system in *Dehalogenimonas* and identify the specific environmental or redox cues that induce *rdhA* transcription. These insights will be essential for better predicting and stimulating *Dehalogenimonas* activity *in situ* at contaminated sites.

### Evidence of *dcpA* dissemination mediated by mobile genetic elements

Following the described procedure (47), we identified a 23-kbp genomic island carrying the *dcpAB* operon in strain NSZ-14 and strain IP3-3. As shown in Fig. 4, this nearly identical mobile region shared by the two strains shows high synteny and is enriched in *rdh* genes, including *dcpA*, *dcpB*, *rdhP*, *rdhS*, and those involved in DNA recombination and replication (e.g., genes coding for recombinases, DNA primase, and RNR). It was detected based on pronounced compositional bias relative to the genome background, including dinucleotide-frequency deviation identified by IslandPath-DIMOB4 (48), and motif-based sequence bias detected by Alien-Hunter (49). In both genomes, these mobile genetic elements lie immediately downstream of a tmRNA-encoding gene (i.e., *ssrA*), a well-known integration hotspot (50), and are flanked by direct repeats (DR), with two DR pairs of 25 bp and 16 bp in strain NSZ-14 and a single 11 bp DR pair in strain IP3-3. The GC content of this mobile region (45.8%) is much lower than the genome averages of strain NSZ-14 (54.0%) and strain IP3-3 (55.9%). In addition, a substantial fraction of genes within this mobile region (6 out of 20) lack functional annotation, which is another hallmark of genomic islands. The conserved synteny of these mobile elements, together with the lack of synteny in the flanking chromosomal regions, suggests that *dcpA* was acquired via horizontal transfer after the divergence of *D. formicexedens* and *D. alkenigignens*. Horizontal acquisition of *dcpA* (locus # ABV300_07400) was also noted in strain 4OHTPN, where it is located downstream of *ssrA* and adjacent to recombinase genes; however, a clear boundary characteristic (i.e., DR) that defines a discrete mobile element could not be detected. In contrast, *dcpA* in other *Dehalogenimonas* strains does not exhibit a comparable genomic context based on synteny and gene-neighborhood comparisons. The genome-to-genome variation in the *dcpA* gene neighborhood indicates recurrent, lineage-specific gene transfer events. Together, these observations provide evidence that horizontal transfer has contributed to *dcpA* acquisition in some *Dehalogenimonas* lineages, but this mechanism alone cannot account for the genus-wide distribution of *dcpA*. Notably, strain NSZ-14 and strain IP3-3 have a common origin, as both were isolated from groundwater at a U.S. Superfund site impacted by a variety of chlorinated alkanes (8, 9). Such contamination settings may confer a selective advantage on dihaloeliminating OHRB, favoring the persistence of *dcpA*-carrying mobile elements, and eventually, the spread of this *rdhA* among co-occurring *Dehalogenimonas* populations.

To elucidate the evolutionary forces acting on *dcpA*, we conducted selective pressure analyses to pinpoint amino acid sites under potential natural selection. Site-level selection was assessed using three methods: MEME (mixed-effects model of evolution), FUBAR (fast unbiased Bayesian approximation), and SLAC (single-likelihood ancestor counting). Each intact *rdhA* sequence encodes a twin-arginine translocation system signal motif and a catalytic dehalogenase (RDH) domain (NCBIFAM TIGR02486). The data set for analysis (Data Set S3) comprises 7 full-length, genome-extracted *dcpA* sequences (1,449–1,464 bp) and 33 partial environmental *dcpA* sequences (1,071–1,086 bp) obtained using *dcpA*-targeted PCR amplicon sequencing (17). Positions 465–1,553 of the multiple sequence alignment, corresponding to the region encoding the RDH domain (i.e., codons 160–468), were retained for analysis. As listed in Table 2, MEME identified 29 of the 487 codon sites as being under episodic positive selection, including 15 within the RDH domain, suggesting the contribution of adaptive evolution to *dcpA* sequence variation. FUBAR and SLAC respectively reported 93 and 42 codon sites under pervasive negative selection, and no site was inferred to be under pervasive positive selection. A total of 11 codon sites in the RDH domain were identified as being under pervasive negative selection by both the FUBAR and SLAC methods. Figure S5 illustrates the difference between non-synonymous substitution rate (dN) and synonymous substitution rate (dS) across *dcpA* codon sites. Raw outputs are reported in Data Set S3. Together, these results suggest that *dcpA* has been shaped by a combination of episodic positive selection, which promotes beneficial variants such as those broadening substrate range, and pervasive purifying selection, which removes deleterious mutations and preserves core enzymatic function. This evolutionary pattern is consistent with the widespread distribution of *dcpA* across *Dehalogenimonas* lineages and the conserved role of DcpA as an RDase catalyzing the dihaloelimination of various C2–C3 alkanes.

**TABLE 2:** Summary of codon-based analyses of *dcpA* sequences using the MEME, FUBAR, and SLAC methods

	Episodic selection	Pervasive selection	
	MEME	FUBAR	SLAC
Number of sites under selection	29	93 (negative) 0 (positive)	42 (negative) 0 (positive)
Number of sites in RDH domain under selection	15 (5%)	54 (17.5%)	16 (5.2%)
Sites in RDH domain under selection*^a^*	225, 283, 287, 302, 317, 328, 343, 355, 359, 372, 377, 392, 405, 434, 448	168, 170, 174, 185, 196, 227, 228, 243, 294, 323, 450	

^*a*^ Codon sites detected by both FUBAR and SLAC are listed and defined as pervasive selection.

### Conclusions

This study reveals that *Dehalogenimonas* spp. have undergone long-term genome streamlining, an evolutionary route to metabolic specialization. Their highly reduced genomes exhibit a pattern in which nonessential functions are discarded, whereas core respiration-associated modules are preferentially retained or expanded to sustain organohalide respiration. *Dehalogenimonas* genomes display a high degree of plasticity with localized variability in gene content, demonstrating tolerance for structural changes in genome organization. Mobile genetic elements are involved in horizontal transfer of certain *rdhA*, facilitating the spread of dehalogenation capacity among *Dehalogenimonas*. The *dcpA* gene, which is broadly distributed across *Dehalogenimonas* lineages, likely represents an ancestral respiratory component within the class *Dehalococcoidia*. Overall, this work offers insights into lineage diversification and niche adaptation in *Dehalogenimonas*.

## MATERIALS AND METHODS

### Genome accession numbers

The GenBank accession numbers for the analyzed *Dehalogenimonas* genomes are NZ_CP058566 (strain GP), NZ_CP018258 (strain NSZ-14), CP002084 (strain BL-DC-9), NZ_LFDV00000000 (strain IP3-3), NZ_CP159307 (strain 4OHTPN), NZ_QEFQ00000000 (strain BRE15M), NZ_CP146612 (strain W), CP011392 (strain WBC-2), and NZ_JBDLLU000000000 (strain THU2). The reference *Dhc* genomes correspond to NC_002936 (strain 195) and NC_007356 (strain CBDB1).

### Orthologous and collinearity analyses

A total of nine publicly available *Dehalogenimonas* genomes were downloaded from GenBank, of which eight were selected for pangenome construction. Annotated protein-coding sequences were used for ortholog analysis using OrthoMCL with an *E* value cutoff of 10^−5^, and for collinearity analysis with MCScanX implemented through the OrthoVenn3 platform using default parameters (51).

### Molecular clock estimation and gene family contractions and expansions

Molecular evolutionary genetics analysis was conducted using MEGA X (52) to predict the divergence times of the *Dehalogenimonas* spp. by the RelTime-ML approach (53) using the TN93+G+I substitution model (Tamura-Nei model with gamma distributed and invariant sites) (54). The full-length 16S rRNA gene sequences retrieved from whole genomes were aligned with Clustal Omega in Geneious software v21.0.4+7-LTS (Biomatters, New Zealand) and then analyzed for phylogeny reconstruction with the neighbor-joining method. The nucleotide sequence alignment and phylogenetic tree were used as the input for RelTime-ML. The divergence time between *D. lykanthroporepellens* and *Dhc* (496 mya) provided by TimeTree (55) was applied to set the divergence time calibration constraint. The estimated divergence times of *Dehalogenimonas* species, inferred from 16S rRNA genes, were then used as input for gene family contraction and expansion analysis with CAFE5 (https://github.com/hahnlab/CAFE5) on OrthoVenn3.

### CDS analysis

*Dehalogenimonas* genomes were re-annotated using the RAST (Rapid Annotation using Subsystem Technology) server (56) and Bakta v1.8.2 (57) with default parameters, and the inferred protein sequences were analyzed with the KEGG database (https://www.kegg.jp) (58) to identify key pathways and modules. The putative enzymes were further curated using the InterPro server (59) to ensure the appropriate length and the presence of known motifs. Multiple alignments of amino acid sequences were performed using Clustal Omega embedded in Geneious v21.0.4+7-LTS. Genome annotation and whole-genome BLAST comparison (60) with BLAST+2.15.0 (0.0001 *E* value cutoff) were visualized in the Proksee (61). Possible genomic islands were predicted by SIGI-HMM and IslandPath-DIMOB that are integrated into IslandViewer 4 (48), and Alien-Hunter (49) in the Proksee platform.

### Phylogenetic analysis

The maximum-likelihood phylogenetic tree was built using IQ-TREE (62) with automatic model selection provided by ModelFinder (63). Branch support analyses included 1,000 ultrafast bootstrap replicates (64) and single-branch test with SH-aLRT (SH-like approximate likelihood ratio test) (65). The tree with the highest log likelihood was visualized in iTOL (66).

### Selective pressure analysis

The selective pressure analysis of the *dcpA* gene was performed via the Datamonkey web application using HyPhy v2.5.68 (67). Positive/diversifying selection is defined for sites with a statistically significant ratio of non-synonymous (dN) to synonymous (dS) substitutions, *ω* > 1, negative/purifying selection is inferred for *ω* < 1, and neutrality is *ω* = 1 (68). Separate analyses were conducted with three site-level selection methods, MEME (mixed-effects model of evolution), FUBAR (fast unbiased Bayesian approximation), and SLAC (single-likelihood ancestor counting). The MEME tests for episodic (at a subset of branches) positive or negative selection and is the preferred approach for detecting positively selected sites (*P* ≤ 0.1). The analysis in FUBAR (with a posterior probability of 0.95) and SLAC (*P* ≤ 0.1) estimates sites subject to pervasive (across the whole phylogeny) positive or negative selection; the sites supported by both methods were defined as pervasive selection sites.

## SUPPLEMENTAL MATERIAL

Data Set S1 aem.00579-26-s0001.xlsx Orthogroup collinearity.

Data Set S2 aem.00579-26-s0002.xlsx Expansion and contraction families.

Data Set S3 aem.00579-26-s0003.xlsx Site-level selection.

Supplemental material aem.00579-26-s0004.docx Figures S1 to S5; Tables S1 to S5.

ASM does not own the copyrights to Supplemental Material that may be linked to, or accessed through, an article. The authors have granted ASM a non-exclusive, world-wide license to publish the Supplemental Material files. Please contact the corresponding author directly for reuse.
