# Supplementary material for: Comparative genomics reveals Dehalogenimonas genome dynamics and evolutionary trajectories toward organohalide respiration
Source: Appl Environ Microbiol. 2026 Jun 3;92(7):e00579-26. doi: 10.1128/aem.00579-26 (PMC13390414; doi:10.1128/aem.00579-26)
Supplement: Supplemental material — Figures S1 to S5; Tables S1 to S5. [file aem.00579-26-s0004.docx]

**Supplementary Information for**

Comparative genomics reveals *Dehalogenimonas* genome dynamics and evolutionary trajectories toward organohalide respiration

Yiru Cui, Xiuying Li, Xiaocui Li, Jingjing Wang, Huijuan Jin, Ke Shi, Jun Yan

Correspondence:

Jun Yan, Key Laboratory of Pollution Ecology and Environmental Engineering, Institute of Applied Ecology, Chinese Academy of Sciences, Shenyang, Liaoning 110016, China, Phone: +86-24-83970426, E-mail: junyan@iae.ac.cn

This PDF file includes:

Figures S1 to S5

Tables S1 to S5

References

**Figure S1**. Numbers of overlapping orthologous gene clusters across *Dehalogenimonas* genomes. Green and gray blocks indicate the presence and absence, respectively, of orthologues gene clusters in each genome.

**Figure S2**. Pairwise heatmap showing the numbers of orthologous protein-coding genes among selected *Dehalogenimonas* strains.

**Figure S3.** Phylogenetic tree and estimated divergence times of representitive OHRB inferred using TimeTree with default settings (www.timetree.org).

**
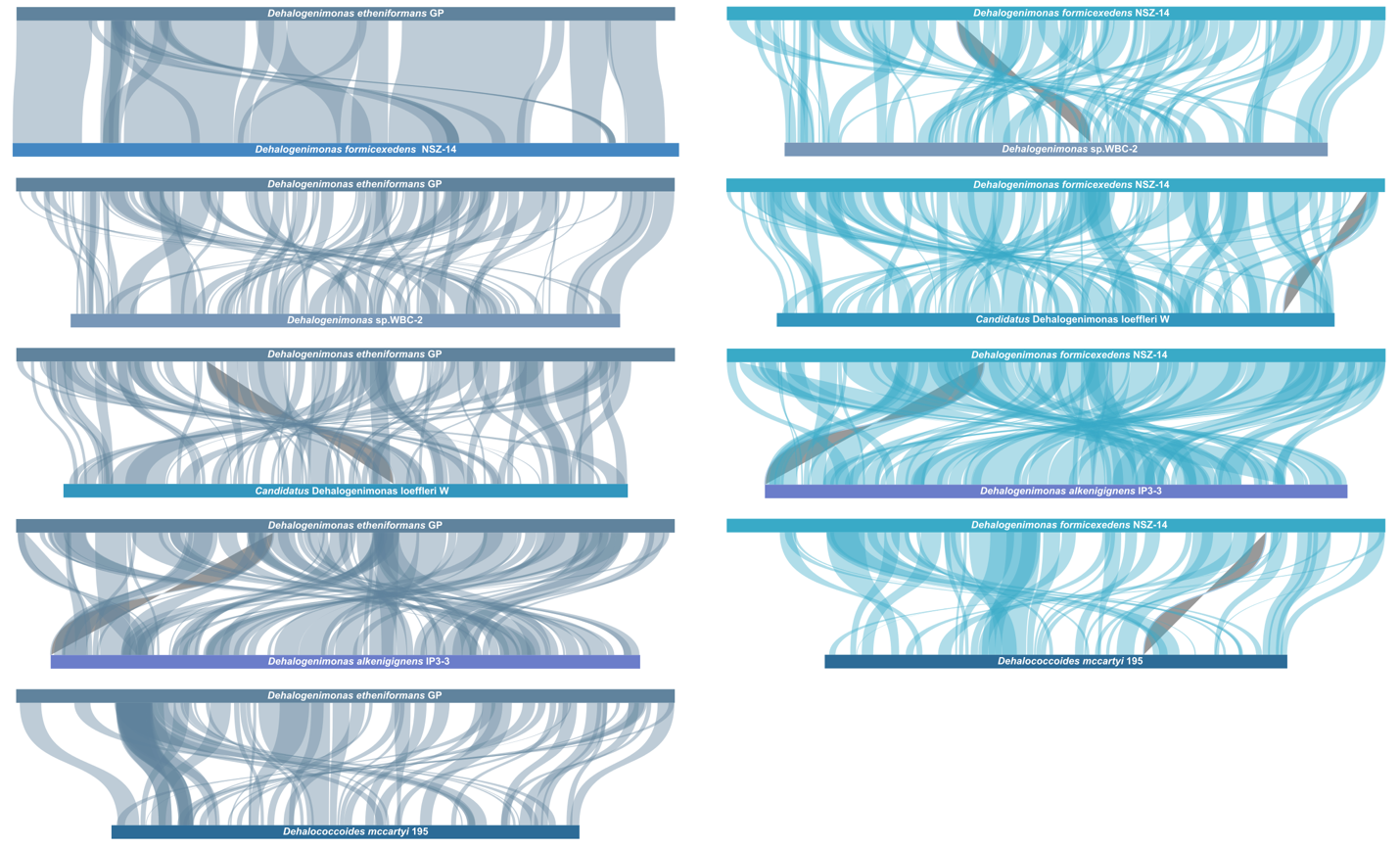
**

**Figure S4.** Pairwise genome collinearity among *Dehalogenimonas* spp. Left, collinearity between the genome of *D. etheniformans* GP and those of selected strains, including *D. formicexedens* NSZ-14, *Dehalogenimonas* sp. WBC-2, ‘*Candidatus* Dehalogenimonas loeffleri’ W, *D. alkenigignens* IP3-3, and *Dhc* strain 195. Right, collinearity between the genome of *D. formicexedens* NSZ-14 and those of other *Dehalogenimonas* strains and *Dhc* strain 195. For strain W, the genome start position was adjusted to the 260-bp position upstream of the chromosomal replication initiator *dnaA* gene. The dark gray region indicates reverse alignment.


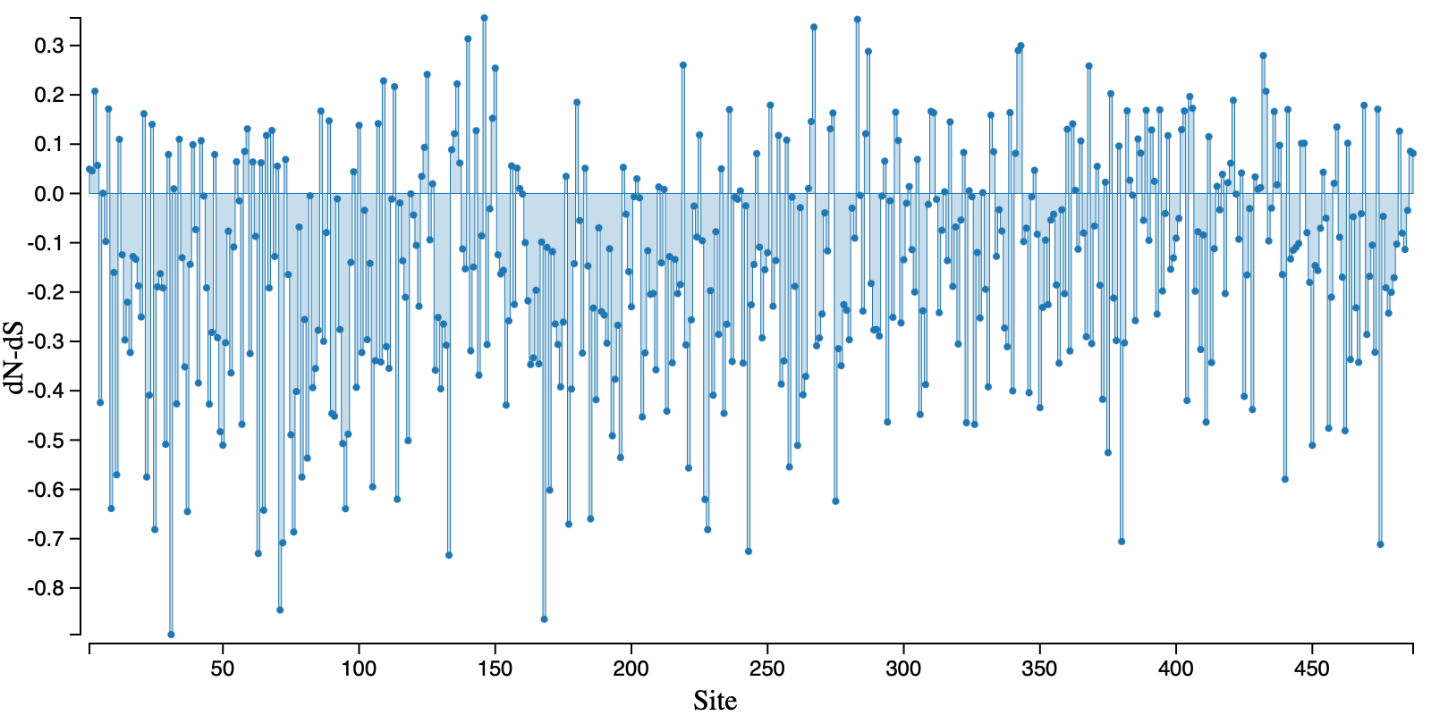


**Figure S5.** Site-by-site SLAC (single-likelihood ancestor counting) analysis of *dcpA* showing the difference between the nonsynonymous substitution rate (dN) and the synonymous substitution rate (dS) at a specific codon site.

**Table S1.** Predicted oxidoreductase complex in *Dehalogenimonas* genomes.

| Oxidoreductase ^a^ | Strain | Gene locus ^b^ | Putative cellular location |
| --- | --- | --- | --- |
| NiFe hydrogenase (Hup, 1) | GP  NSZ-14  IP3-3  BRE15M  4OHTPN  W  THU2  WBC-2  BL-DC-9 | HX448_00340-00350  Dform_01917-01919  DEALK_07970-07990  DD509_07095-07105  ABV300_09090-09100  V8247_04695-04705  ABFB09_05210-05225  DGWBC_0126-0128  Dehly_0025-0027 | Membrane-bound  periplasmic |
| NiFe hydrogenase (Vhu type, 3) and CoB-CoM heterodisulfide reductase  (Vhu-Hdr complex) | GP  NSZ-14  IP3-3  BRE15M  4OHTPN  W  THU2  WBC-2  BL-DC-9 | HX448_06310-06345  Dform_00936-00943  DEALK_14240-14310  DD509_01365-01400  ABV300_05895-05930  V8247_08070-08105  ABFB09_02270-02305  DGWBC_1413-1420  Dehly_0725-0732 | Soluble cytoplasmic |
| Cofactor F_420_-reducing NiFe hydrogenase homolog (3) ^c^ | GP  NSZ-14  IP3-3  BRE15M  4OHTPN  W  THU2  WBC-2  BL-DC-9 | HX448_04540-04550  Dform_00600-00602  DEALK_17650-17670  DD509_08165-08175  ABV300_04350-04360  V8247_00485-00495  ABFB09_07475-07485  DGWBC_1202-1204  Dehly_0927-0929 | Soluble cytoplasmic |
| NiFe hydrogenase | GP  NSZ-14  IP3-3  BRE15M  4OHTPN  W  THU2  WBC-2  BL-DC-9 | HX448_00425-00435  Dform_01922-01924  DEALK_18060-18090  DD509_07950-07965  ABV300_04135-04150  V8247_02395-02410  ABFB09_01155-01170  DGWBC_0298-0301  Dehly_1286-1289 | Soluble cytoplasmic |
| NiFe hydrogenase (Hyc, 4) | GP  NSZ-14  IP3-3  BRE15M  4OHTPN  W  THU2  WBC-2 | HX448_10295-10320  Dform_01861-01866  DEALK_08540-08590  DD509_02940-02965  ABV300_08815-08840  V8247_04875-04900  ABFB09_09275-09300  DGWBC_1667-1672 | Membrane-bound  cytoplasmic |
| NiFe hydrogenase (Ech, 4) | GP | HX448_04330-04370 | Membrane-bound  cytoplasmic |
| NADH dehydrogenase  (Complex I, NuoABCDHIJKLMN) | GP  NSZ-14  IP3-3  BRE15M  4OHTPN  W  THU2  WBC-2  BL-DC-9 | HX448_05470-05520  Dform_00743-00753  DEALK_14940-15040  DD509_00985-01035  ABV300_06260-06310  V8247_09050-09100  ABFB09_04860-04910  DGWBC_0934-0944  Dehly_0831-0841 | Membrane-bound  cytoplasmic |
| Formate dehydrogenase N (Fdn) | GP  NSZ-14  IP3-3  BRE15M  4OHTPN  W  THU2 | HX448_03695-03705  Dform_00419-00421  DEALK_19090-19115  DD509_05300-05305 ^d^  ABV300_03865-03875  V8247_04725-04735  ABFB09_01720-01730 | Membrane-bound  periplasmic |
| Pyruvate-ferredoxin oxidoreductase (Por) | GP  NSZ-14  IP3-3  BRE15M  4OHTPN  W  THU2  WBC-2  BL-DC-9 | HX448_05635-05665  Dform_00778-00784  DEALK_13470-13530  DD509_01770-01800  ABV300_05505-05535  V8247_08860-08890  ABFB09_04080-04110  DGWBC_1025-1031  Dehly_0623-0629 | Soluble cytoplasmic |

^a^ Names and classification numbers from Vignais et al. (1).

^b^ Locus tag is for the predicted catalytic subunit.

^c^ This enzyme also reduces the riboflavin analog of F_420_, flavins and methylviologen.

^d^ Only two subunits were identified.

**Table S2.** Predicted selenocysteine synthesis and insertion *sel* gene cluster in *Dehalogenimonas* genomes.

|  | Strain | Gene locus | tRNA-Sec (*selC*) locus |
| --- | --- | --- | --- |
| 4-gene cluster (*selCDAB*) | GP  4OHTPN  W  THU2  WBC-2  BL-DC-9 | HX448_01345-01360  ABV300_01165-01180  V8247_02615-02630  ABFB09_06335-06350  DGWBC_1761-1764  Dehly_R0051, Dehly_1500-1502 | HX448_01345  ABV300_01165  V8247_02630  ABFB09_06335  DGWBC_1761  Dehly_R0051 |
| 5-gene cluster  (*selC*-intervening CDS-*selDAB*) | NSZ-14  BRE15M  IP3-3 | Dform_02176-02180  DD509_05255-05275  DEALK_04950-04975, DEALK_t00110 (old locus tag)  DEALK_RS02410-RS02420, DEALK_RS09740,  DEALK_RS02425 (new locus tag) | Dform_02176  DD509_05255  DEALK_t00110 (old locus tag)  DEALK_RS02425 (new locus tag) |

**Table S3.** Summary of the numbers of CDSs, CDSs assigned to homologous families or singletons, and homologous gene families in each analyzed Dehalogenimonas genome.

|  | GP^T^ | NSZ-14^T^ | IP3-3^T^ | 4OHTPN | BRE15M | W | WBC-2 | THU2 |
| --- | --- | --- | --- | --- | --- | --- | --- | --- |
| CDSs | 2,015 | 2,066 | 1,878 | 1,752 | 1,689 | 1,762 | 1,721 | 1,850 |
| CDSs in homologs | 1,708 | 1,832 | 1,777 | 1,667 | 1,627 | 1,548 | 1,426 | 1,663 |
| CDSs in singletons | 307 | 234 | 101 | 85 | 62 | 214 | 295 | 187 |
| Homolog families | 1,641 | 1,745 | 1,726 | 1,617 | 1,616 | 1,706 | 1,381 | 1,599 |

**Table S4.** Pairwise amino acid identity (%) among predicted RdhS histidine kinases. The analysis includes RdhS sequences from *Dehalogenimonas* (strain GP, strain NSZ-14, strain IP3-3 and strain WBC-2), *Dhc* (strain 195 and strain CBDB1), and *Sulfurospirillum multivorans* DSM 12446, based on a multiple sequence alignment. Corresponding gene locus tags are provided.

|  | GP | NSZ-14 | IP3-3 | WBC-2 | 195 | CBDB1 | DSM 12446 |
| --- | --- | --- | --- | --- | --- | --- | --- |
| GP (HX448_10000) |  | 33.096 | 33.096 | 100 | 23.902 | 15.789 | 11.149 |
| NSZ-14 (Dform_01457) | 33.10 |  | 100 | 33.607 | 21.546 | 16.522 | 12.199 |
| IP3-3 (DEALK_17140) | 33.10 | 100 |  | 33.607 | 21.546 | 16.522 | 12.199 |
| WBC-2 (DGWBC_0415) | 100 | 33.607 | 33.607 |  | 23.229 | 15.854 | 11.149 |
| 195 (DET0315) | 23.90 | 21.546 | 21.546 | 23.229 |  | 16.958 | 13.043 |
| CBDB1 (cbdbA82) | 15.79 | 16.522 | 16.522 | 15.854 | 16.958 |  | 11.404 |
| DSM 12446 (SMUL_1534) | 11.15 | 12.199 | 12.199 | 11.149 | 13.043 | 11.404 |  |

**Table S5.** Pairwise amino acid identity (%) of predicted RdhP response regulators. The analysis includes RdhP sequences from *Dehalogenimonas* (strain GP, strain NSZ-14, strain IP3-3, strain WBC-2), *Dhc* (strain 195, strain CBDB1), and *Sulfurospirillum multivorans* DSM 12446, based on a multiple sequence alignment. Corresponding gene locus tags are provided.

|  | GP RdhP_1 | GP  RdhP_2 | NSZ-14 | IP3-3 | WBC-2 | 195 | CBDB1 | DSM 12446 |
| --- | --- | --- | --- | --- | --- | --- | --- | --- |
| GP RdhP_1 (HX448_10005) | 34.07 |  | 44.69 | 44.69 | 33.18 | 36.73 | 34.2 | 24.34 |
| GP RdhP_2 (HX448_10010) | 34.07 |  | 35.53 | 35.53 | 99.11 | 33.63 | 28.02 | 24.56 |
| NSZ-14 (Dform_01459) | 44.69 | 35.53 |  | 100 | 35.11 | 39.38 | 27.59 | 23.25 |
| IP3-3 (DEALK_17160) | 44.69 | 35.53 | 100 |  | 35.11 | 39.38 | 27.59 | 23.25 |
| WBC-2 (DGWBC_0413) | 33.18 | 99.11 | 35.11 | 35.11 |  | 33.63 | 27.51 | 24.00 |
| 195 (DET0316) | 36.73 | 33.63 | 39.38 | 39.38 | 33.63 |  | 28.14 | 21.59 |
| CBDB1 (cbdbA83) | 34.2 | 28.02 | 27.59 | 27.59 | 27.51 | 28.14 |  | 20.34 |
| DSM 12446 (SMUL_1535) | 24.34 | 24.56 | 23.25 | 23.25 | 24.00 | 21.59 | 20.34 |  |

**References**

1. Vignais PM, Billoud B. 2007. Occurrence, classification, and biological function of hydrogenases: An overview. Chem Rev 107:4206-4272.
